# Atypical presentation and diagnosis of a metastatic grade II pancreatic neuroendocrine tumor in a 52-year-old female patient: A case report

**DOI:** 10.3892/mi.2025.271

**Published:** 2025-09-26

**Authors:** Luisa Fernanda Montemayor Burrola, Hugo Alberto Roblero López, América Villalobos Ulate, Edgar Iván Martínez Rosales

**Affiliations:** Department of Internal Medicine, General Regional Hospital No. 1, Mexican Institute of Social Security (IMSS), Chihuahua, Chihuahua 31000, Mexico

**Keywords:** pancreatic neuroendocrine tumor, atypical presentation, syncope, neuroendocrine tumor diagnosis, constitutional symptoms

## Abstract

Pancreatic neuroendocrine tumors (PanNETs) are rare malignant neoplasms characterized by slow growth and variable clinical presentation. Although some secrete functional hormones, the majority remain clinically silent or manifest with non-specific symptoms, posing a challenge to early diagnosis. The present study describes the case of a 52-year-old female patient with a significant family history of malignancy, who presented with a syncopal episode following months of weight loss, fatigue and vague abdominal symptoms. A diagnostic workup revealed severe anemia and imaging findings consistent with metastatic disease. A computed tomogrpaphy-guided liver biopsy confirmed a moderately differentiated, grade II neuroendocrine tumor. Treatment with intramuscular octreotide was initiated, and the patient was discharged under palliative care. The case described herein underscores the clinical complexity and often subtle nature of PanNETs, and highlights the importance of considering them in the differential diagnosis of persistent constitutional symptoms, particularly in high-risk individuals. Timely diagnosis supported by histopathology and immunohistochemistry, combined with access to targeted therapies, is essential to improve the outcomes of patients. The integration of molecular screening and emerging therapeutic targets may transform the management of PanNETs, enabling earlier and more personalized interventions in the near future.

## Introduction

Pancreatic neuroendocrine tumors (PanNETs) are a group of malignant neoplasms that arise exclusively from the neuroendocrine tissue of the pancreas. They are typically slow-growing and exhibit the strong expression of immunohistochemical markers, such as synaptophysin and chromogranin A (1,2). PanNETs account for ~2 to 7% of all pancreatic tumors (3) and include entities, such as insulinoma, gastrinoma, VIPoma, ACTHoma, PPoma and glucagonoma, among others. These neoplasms may originate from either differentiated pancreatic cells or stem cells located within the islets of Langerhans (4).

The incidence of PanNETs has exhibited a steady increase worldwide, with notable regional and national variations. The average reported incidence is ~1 case per 100,000 inhabitants (5,6). According to the World Health Organization (WHO), PanNETs are classified based on histological differentiation and molecular expression profiles (7). Well-differentiated PanNETs exhibit a low proliferative activity, whereas poorly differentiated tumors are associated with high Ki-67 indices and demonstrate a more aggressive clinical behavior (8). Regardless of grade, these neoplasms often present with eosinophilic cytology, hyperchromatic nuclei, and a fibrotic stroma typically devoid of necrosis (9).

Approximately 30% of PanNETs secrete functional hormones, which may facilitate clinical recognition through symptoms related to ectopic hormone production (10,11). However, the majority of PanNETs are clinically silent or non-specific, necessitating a comprehensive diagnostic approach involving biochemical markers, molecular analysis and advanced imaging modalities (12,13). In this context, genetic screening aimed at detecting specific pathogenic mutations has also been proposed (14). The treatment of PanNETs is guided by their malignant potential and functional status. Poorly differentiated tumors usually require aggressive treatment strategies due to their unfavorable prognosis (15). By contrast, well-differentiated functional tumors may be initially managed with active surveillance, with surgical resection and lymphadenectomy indicated upon evidence of disease progression (16,17).

Early detection, close clinical monitoring and timely surgical intervention are associated with improved survival outcomes of patients with PanNETs (18). Nevertheless, despite the increasing global incidence, the majority of cases continue to be diagnosed at advanced stages, thus often being associated with a poor prognosis (19).

Considering the above, the present study describes the clinical case of a patient with a confirmed diagnosis of a functional PanNET whose initial presentation was atypical. Given the rarity of these tumors and their often non-specific manifestations, the present case report aimed to highlight key clinical features that may support early diagnostic suspicion in patients presenting with compatible symptoms, thereby contributing to the prompt recognition of these uncommon neoplasms.

## Case report

A 52-year-old female patient presented at Hospital General Regional No. 1, Unidad Morelos of the Instituto Mexicano del Seguro Social (IMSS), Chihuahua, Mexico, with a family history of malignancies, including colorectal, breast, pancreatic cancer, and an unspecified lymphoma. She had no history of chronic diseases or substance abuse. Her surgical history included an appendectomy at age 21, a myomectomy at age 42 and a hysterectomy at age 45.

She was admitted to the emergency department at Hospital General Regional No. 1, Unidad Morelos of the Instituto Mexicano del Seguro Social (IMSS) following an episode of syncope, with no other associated symptoms. During a clinical evaluation, she reported the onset of symptoms with unintentional weight loss of ~25% of her usual body weight over a 6-month period (from 78 to 58 kg), accompanied by fatigue, generalized weakness, and hyporexia secondary to cheilitis and glossitis. This was followed by intermittent colicky abdominal pain localized to the right upper quadrant and mesogastric region, with no radiation or identifiable triggering or relieving factors. More recently, she reported the worsening of her clinical condition with progressive dyspnea initially upon moderate exertion and later minimal exertion, as well as orthopnea and predominantly daytime lower extremity edema.

Upon a physical examination, altered vital signs were recorded: A blood pressure of 96/58 mmHg, a heart rate of 115 beats per minute and a respiratory rate of 25 breaths per minute. Laboratory analyses revealed normocytic normochromic anemia with a hemoglobin level of 6.8 g/dl (reference range, 12-15.5 g/dl), a mean corpuscular volume of 80.4 fl (reference range, 80-96 fl), mean corpuscular hemoglobin level of 25.2 pg (reference range, 27-33 pg) and a mean corpuscular hemoglobin concentration of 31.3 g/dl (reference range, 32-36 g/dl). The patient was subsequently admitted to the internal medicine ward for further diagnostic workup.

**Figure 1:**
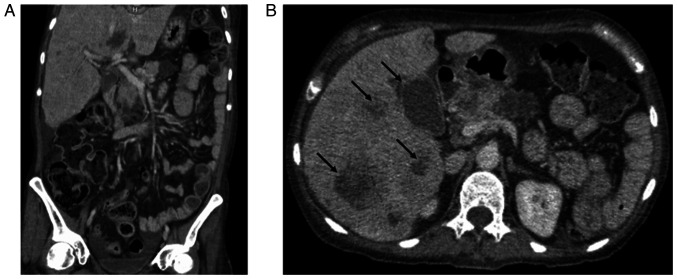
Contrast-enhanced abdominal and pelvic computed tomography scan. (A) Coronal view illustrating areas of fat stranding in the perihepatic region, compatible with fluid overload. (B) Axial view demonstrates heterogeneous liver parenchyma due to multiple isodense lesions with central hypodensity suggestive of necrosis (indicated by black arrows). Following contrast administration, the lesions exhibited homogeneous centripetal enhancement. No retroperitoneal lymphadenopathy or lesions were observed.

A contrast-enhanced computed tomography (CT) scan of the abdomen and pelvis revealed multiple hepatic and pancreatic lesions of varying lengths and isodense characteristics, suggestive of metastatic disease, as well as hydrocholecystis (Fig. 1). Given these findings, a CT-guided liver biopsy was performed. A histopathological examination was performed using hematoxylin and eosin (H&E) staining (Merck KGaA). The surgical specimens were fixed in 10% neutral-buffered formalin at room temperature for 24 h, embedded in paraffin, and sectioned at a thickness of 4 µm. The sections were subsequently deparaffinized, rehydrated, and stained with hematoxylin for 5 min, followed by eosin counterstaining for 2 min. The slides were mounted with a synthetic resin and examined under a light microscope (Olympus CX43, Olympus Corporation) at x40 magnification.

**Figure 2:**
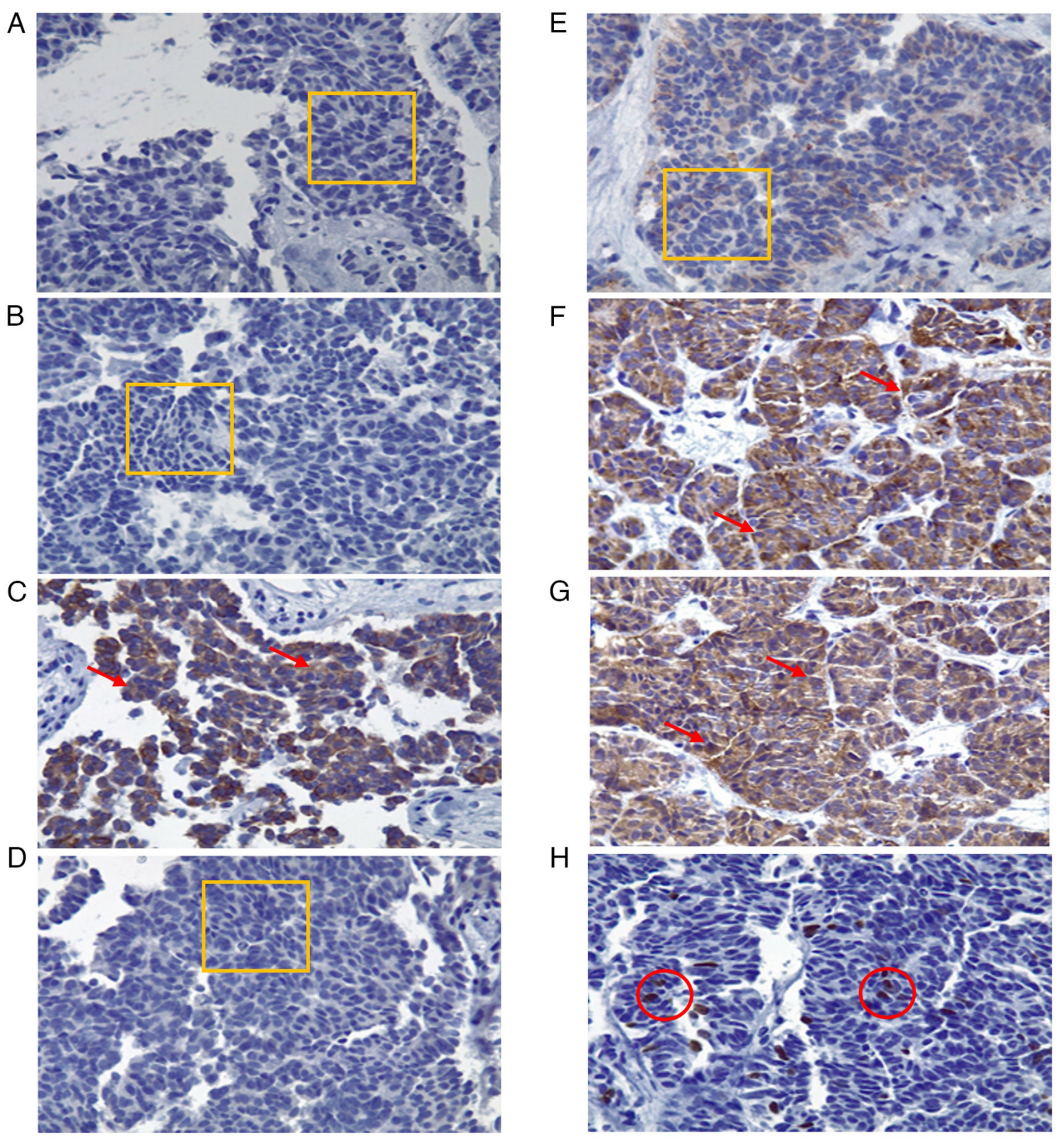
Immunohistochemical analysis of the biopsied tissue (magnification, x40). Histological specimens illustrating neoplastic cells with cylindrical and cuboidal morphology, irregular nuclear enlargement, inconspicuous nucleoli and scant cytoplasm. Three mitotic figures per high-power field (40X objective) are identified. Findings are consistent with moderately differentiated adenocarcinoma associated with extensive desmoplasia. (A) CK7 immunohistochemistry: 0% expression, negative intensity. (B) CK20: 0% expression, negative intensity. (C) CK19: 90% of cells positive, 80% intensity, cytoplasmic pattern. (D) MUC5AC: 0% expression, negative intensity. (E) CA 19-9: 0% expression, negative intensity. (F) Chromogranin A: 100% expression, 90% intensity, cytoplasmic pattern. (G) Synaptophysin: 100% expression, 90% intensity, cytoplasmic pattern. (H) Ki-67: proliferation index of 8%, 100% intensity, nuclear pattern. (A, B, D and E) The thin orange rectangle outlines the evaluated area with no staining (negative expression). C, F and G) Red arrows indicate cells with brown cytoplasmic staining (positive cytoplasmic pattern). (H) Thin red circles indicate nuclei with brown nuclear staining (positive nuclear pattern, 8%).

The histopathological examination revealed a malignant epithelial neoplasm with focal areas of both recent and chronic coagulative necrosis. The lesion exhibited fibrotic areas and was composed of cellular clusters with cylindrical or cuboidal morphology, nuclear enlargement, oval to irregular or elongated nuclei, inconspicuous nucleoli and scant cytoplasm. Immunohistochemical analysis was performed on paraffin-embedded tissue blocks using the avidin-biotin immunoenzymatic method at the Pathology and Immunohistochemistry Laboratory of the Hospital General Regional No. 1, Unidad Morelos of the Instituto Mexicano del Seguro Social (IMSS). Sections of 4 µm thickness were obtained from formalin-fixed paraffin-embedded tissue. For intracellular antigens, permeabilization was carried out with 0.1% Triton X-100 (Merck KGaA) for 10 min at room temperature. Endogenous peroxidase activity was blocked with 3% hydrogen peroxide for 10 min, followed by blocking with 5% normal goat serum (MilliporeSigma) for 30 min at room temperature. The following primary antibodies were used: CK7 (1:100, cat. no. M7018, Dako, Agilent Technologies, Inc.), CK20 (1:100, cat. no. M7019, Dako, Agilent Technologies, Inc.), CK19 (1:200, cat. no. ab15463, Abcam), MUC5AC (1:100, cat. no. ab3649, Abcam), CA19-9 (1:100, cat. no. ab15146, Abcam), chromogranin A (1:200, cat. no. M0869, Dako, Agilent Technologies, Inc.), synaptophysin (1:200, cat. no. M0776, Dako), and Ki-67 (1:200, cat. no. M7240, Dako, Agilent Technologies, Inc.). Incubation with primary antibodies was performed overnight at 4˚C. Sections were then incubated with secondary antibodies conjugated to horseradish peroxidase (HRP) (EnVision+ System-HRP, Dako, Agilent Technologies, Inc.) for 30 mins at room temperature. Immunoreactivity was visualized using 3,3'-diaminobenzidine (DAB; Dako, Agilent Technologies, Inc.) as a chromogen, followed by counterstaining with hematoxylin for 1 min at room temperature. Slides were mounted with resin and examined using a light microscope (Olympus CX43, Olympus Corporation) at x40 magnification. Immunohistochemical analyses confirmed the diagnosis of a grade II neuroendocrine tumor (Fig. 2). The patient was evaluated by the medical oncology team, who recommended initiating treatment with intramuscular octreotide at a dose of 20 mg every 21 days. She was discharged under palliative care with close outpatient follow-up.

At her first outpatient visit on January 24, 2025, the laboratory results revealed hemoglobin levels at 11.3 g/dl, a platelet count of 662x10^9^/l and a serum albumin level of 2.5 g/dl. Despite initial clinical stability and an ECOG performance status of 1, the patient began experiencing persistent vomiting and progressive weight loss. By March 7, 2025, laboratory tests revealed a decline in hemoglobin levels to 9.3 g/dl and a platelet count of 412x10^9^/l. She was hospitalized again on April 9, 2025, due to a worsening functional status, but was discharged the same day under symptomatic management. At the final follow-up, at 6 months after discharge, the patient remained alive under palliative care, with no major complications other than general functional decline.

## Discussion

The present study describes the clinical case of a middle-aged female patient who, following a prolonged course of non-specific constitutional symptoms, was diagnosed with a grade II, moderately differentiated, metastatic PanNET. The case described herein underscores the clinical complexity and frequently indolent nature of these neoplasms, which often leads to delayed diagnosis despite evidence of systemic progression.

Previous case reports have described PanNETs with atypical clinical presentations similar to the present case, in which non-specific constitutional symptoms preceded definitive diagnosis by several months (20). In particular, incidental findings or vague symptoms, such as weight loss, fatigue, or syncope have led to the eventual discovery of PanNETs, emphasizing the need for a more thorough evaluation in patients with persistent unexplained symptoms (21). The clinical value of this case lies in highlighting how the absence of classic signs can delay the diagnosis of potentially treatable neoplasms, particularly in individuals with a significant family history of malignancy (22). It also underscores the importance of including PanNETs in the differential diagnosis in high-risk individuals, even in the absence of functional signs (23). Furthermore, the present case points to the need for future research evaluating the role of genetic screening in predisposed populations and the use of molecular biomarkers as tools for early detection and non-invasive monitoring of neuroendocrine tumors.

Globally, neuroendocrine neoplasms account for only 0.5% of all tumors, with ~70% of them located in the gastrointestinal tract and pancreas (24). The pancreas is a dual-function organ in which endocrine and exocrine components interact through shared molecular mechanisms under both physiological and pathological conditions, including tumorigenesis (25). In the case of PanNETs, their clinical progression is often slower and less symptomatic compared to other pancreatic neoplasms, rendering early detection more challenging (26).

In this event, the absence of classic clinical signs such as jaundice, severe abdominal pain, or symptoms related to hormonal hypersecretion suggests a non-functional PanNET. These tumors are often diagnosed incidentally or when they cause compressive or vague systemic symptoms (27). In the case described herein, it was a syncopal episode that prompted medical attention and ultimately led to the diagnosis, despite the presence of prior symptoms consistent with constitutional syndrome.

Several studies have reported an increase in incidental diagnoses of PanNETs in individuals without an apparent oncological history, particularly in those presenting with long-standing digestive symptoms (28). In the case in the present study, diagnostic confirmation was achieved through liver biopsy and immunohistochemistry, demonstating positivity for characteristic neuroendocrine tumor markers and a Ki-67 index consistent with a grade II neoplasm, according to the current WHO classification (29,30).

The prognosis of metastatic PanNETs is variable, with 3-year survival rates ranging from 13 to 54% (31). This variability is closely related to tumor burden, functional status and the availability of therapeutic interventions. In the patient in the present study, treatment was initiated with a somatostatin analog (octreotide), which is considered first-line therapy for intermediate-grade PanNETs, although the efficacy of this approach in advanced stages remains limited (32).

Moreover, the significant family history of malignancy of the patient raises the possibility of an underlying genetic predisposition. This highlights the importance of implementing targeted screening strategies in individuals with a family history of PanNETs or other endocrine neoplasms. Recent research has proposed the use of molecular biomarkers and liquid biopsy as promising tools for early detection and non-invasive monitoring of these tumors in high-risk populations (33).

Currently, new molecular targets are under investigation for the treatment of PanNETs resistant to conventional therapies. Among these, FOXM1 (implicated in multiple oncogenic processes) has been identified (34), along with CYR61, a tumor-promoting gene, and the proteins, PAK4 and NAMPT, whose overexpression in patient biopsies suggests their potential as therapeutic targets in advanced disease stages (35). These research pathways may lead to the timely development of more effectively targeted therapies, even in patients with tumor recurrence (36).

The atypical presentation in the patient in the present study suggests the need for the closer monitoring of individuals with a strong familial history of cancer who present mild or non-specific symptoms, including consideration of proactive screening in selected cases. However, as the present case report focuses on a single patient, it does not allow for the generalization regarding the clinical presentation or treatment response of grade II PanNETs. Furthermore, no genetic or molecular profiling was performed in this case, which would have provided greater insight into the etiology and potential hereditary variants involved. Therefore, targeted familial screening is recommended in similar cases.

In conclusion, the present case report highlights the importance of considering PanNETs in the differential diagnosis of persistent and non-specific constitutional symptoms, particularly in patients with a significant family history of malignancy. Timely diagnosis, supported by histopathological and immunohistochemical techniques, along with access to targeted therapies, is essential to improve the prognosis of patients with these neoplasms, which often present with variable clinical behavior. The future integration of molecular screening methods may transform the management of PanNETs, enabling earlier and more personalized interventions.

## Data Availability

The data generated in the present study may be requested from the corresponding author.
